# Surface Proteins and Pneumolysin of Encapsulated and Nonencapsulated *Streptococcus pneumoniae* Mediate Virulence in a Chinchilla Model of Otitis Media

**DOI:** 10.3389/fcimb.2016.00055

**Published:** 2016-05-18

**Authors:** Lance E. Keller, Jessica L. Bradshaw, Haley Pipkins, Larry S. McDaniel

**Affiliations:** Department of Microbiology and Immunology, University of Mississippi Medical CenterJackson, MS, USA

**Keywords:** *Streptococcus pneumoniae*, pneumococcal surface proteins, PspK, nonencapsulated *Streptococcus pneumoniae*, NESp, pneumococcus, chinchilla, otitis media

## Abstract

*Streptococcus pneumoniae* infections result in a range of human diseases and are responsible for almost one million deaths annually. Pneumococcal disease is mediated in part through surface structures and an anti-phagocytic capsule. Recent studies have shown that nonencapsulated *S. pneumoniae* (NESp) make up a significant portion of the pneumococcal population and are able to cause disease. NESp lack some common surface proteins expressed by encapsulated pneumococci, but express surface proteins unique to NESp. A chinchilla model of otitis media (OM) was used to determine the effect various pneumococcal mutations have on pathogenesis in both NESp and encapsulated pneumococci. Epithelial cell adhesion and invasion assays were used to examine the effects in relation to deletion of intrinsic genes or expression of novel genes. A mouse model of colonization was also utilized for comparison of various pneumococcal mutants. It was determined that pneumococcal surface protein K (PspK) and pneumolysin (Ply) affect NESp middle ear pathogenesis, but only PspK affected epithelial cell adhesion. Experiments in an OM model were done with encapsulated strains testing the importance of native virulence factors and treatment of OM. First, a triple deletion of the common virulence factors PspA, PspC, and Ply, (ΔPAC), from an encapsulated background abolished virulence in an OM model while a PspC mutant had detectable, but reduced amounts of recoverable bacteria compared to wildtype. Next, treatment of OM was effective when starting antibiotic treatment within 24 h with resolution by 48 h post-treatment. Expression of NESp-specific virulence factor PspK in an encapsulated strain has not been previously studied, and we showed significantly increased adhesion and invasion of human epithelial cells by pneumococci. Murine colonization was not significantly increased when an encapsulated strain expressed PspK, but colonization was increased when a capsule mutant expressed PspK. The ability of PspK expression to increase colonization in a capsule mutant despite no increase in adhesion can be attributed to other functions of PspK, such as sIgA binding or immune modulation. OM is a substantial economic burden, thus a better understanding of both encapsulated pneumococcal pathogenesis and the emerging pathogen NESp is necessary for effective prevention and treatment.

## Introduction

The pathogenesis of *Streptococcus pneumoniae* (the pneumococcus) is varied and complex due in part to surface structures (Gillespie and Balakrishnan, 2000; Hammerschmidt, 2006; Thornton et al., 2010). Pneumococcal diseases account for ~1 million childhood deaths annually worldwide (O'Brien et al., 2009). However, introduction of the pneumococcal conjugate vaccine (PCV) has led to a dramatic decline in invasive pneumococcal disease (IPD) (Fitzwater et al., 2012). The currently available pneumococcal vaccines target specific pneumococcal polysaccharide serotypes, 23 in Pneumovax (Pneumovax® 23; PPSV23; Merck, Whitehouse Station, NJ, USA), and 13 in Prevnar [Prevnar 13®; PCV13; Pfizer (formerly Wyeth Pharmaceuticals), New York, NY, USA]. Despite extensive use of the PCV in certain parts of the world, noninvasive pneumococcal infections are still prevalent (Weinberger et al., 2011). Noninvasive pneumococcal infections include nonbacteremic pneumonia, conjunctivitis, otitis media (OM), and sinusitis. The pneumococcus is a common etiological agent of OM. In the United States, OM is responsible for most pediatrician visits (McCaig and Hughes, 1995; Gonzales et al., 2001; Lieberthal et al., 2013).

The major pneumococcal virulence factor and target of the PCV is the pneumococcal polysaccharide capsule. There are at least 97 antigenically distinct capsule types (serotypes), as well as pneumococci that do not express any capsule (Geno et al., 2015). Encapsulated strains are dependent on the presence of capsule for all stages of the life cycle, from colonization to virulence, while nonencapsulated *S. pneumoniae* (NESp) do not require a capsule (Kadioglu et al., 2008). Pneumococcal surface proteins are required for colonization and mediate virulence independent of the capsular status. Pneumococcal surface proteins are classified by means of surface attachment and include choline binding proteins (CBPs), LPxTG binding, lipoproteins, and nonclassical surface proteins (Bergmann and Hammerschmidt, 2006).

The surface of NESp and encapsulated pneumococci vary greatly, not only because of the presence or absence of capsule, but also based on the various surface proteins expressed. Regardless of what surface proteins are expressed in either NESp or encapsulated pneumococci, they are essential for colonization and pathogenesis (Valentino et al., 2014). The surface composition between different encapsulated strains also varies, usually as a result in variations of what genes are encoded and expressed, along with different protein isoforms. For instance, the encapsulated pneumococcal CBPs PspA and PspC have multiple variants and have been shown to be important for virulence in both invasive and noninvasive infections (Ogunniyi et al., 2007a). It is important to understand the alterations in virulence profiles as a consequence of differential surface protein expression in order to better understand which strains may be more pathogenic. Furthermore, genetic exchange between pneumococci occurs rapidly and can alter the virulence potential of a strain. Past studies examining alterations in surface proteins have shown that during OM, deletion of PspC reduced virulence while loss of PspA completely eliminated disease in a serotype 2 background (Schachern et al., 2014). NESp lack PspA and PspC but some express the LPxTG binding protein PspK, which has been shown to increase NESp colonization and virulence during OM (Park et al., 2012; Keller et al., 2013, 2014, 2015). We have previously demonstrated adherence to epithelial cells is an important function of NESp surface proteins, and increased adhesion of NESp correlated to increased bacterial burden during OM (Keller et al., 2013, 2014). Another important virulence factor that all known encapsulated and nonencapsulated pneumococci possess is pneumolysin (Ply). It has been shown that Ply has a significant function during infection (Mitchell and Mitchell, 2010). The role of Ply in NESp virulence during OM has not been previously established.

Pneumococci are known to exchange genetic material that has resulted in a high frequency of recombination within the *cps* locus, potentially as a consequence of selective pressure from the use of the PCV (Croucher et al., 2011). Genetic exchange at the *cps* locus between encapsulated pneumococci and NESp may allow encapsulated strains to persist transiently as a nonencapsulated variant despite vaccination. High rates of chromosomal recombination are also focused around regions harboring genes for antibiotic resistance (Croucher et al., 2011). Antibiotic resistance is commonly observed in pneumococcal strains, and NESp often harbor multiple drug resistances (Sulikowska et al., 2004; Chewapreecha et al., 2014; Keller et al., 2016). Due to rapid transfer of resistance genes between pneumococcal strains, prompt, and targeted treatment is necessary for pneumococcal infections. Timely treatment allows for faster resolution and may limit dissemination into other sites.

The current study demonstrates that the absence of Ply reduces the ability of NESp to cause OM. Also, epithelial cell adherence is an important function of OM virulence, but does not directly lead to pathogenesis of encapsulated strains. Additionally, we demonstrate that expression of PspK in capsule mutants partially compensates for the loss of capsule during colonization, thus affording pneumococci a way to circumvent increased use of capsule targeting vaccines.

## Methods

### Bacterial strains

Table 1 contains bacterial strains, relevant mutations, and selective markers used in the current study. All pneumococcal strains were grown at 37°C in 5% CO_2_ in Todd-Hewitt medium with 0.5% yeast extract (THY), or on sheep blood agar (BA) with appropriate antibiotic selection as indicated in Table 1. Genomic and plasmid DNA was obtained using manufacturers protocols with a DNeasy blood and tissue kit (Qiagen) or a plasmid minikit (Qiagen), respectively. Plasmids were maintained in *Escherichia coli* strain DH5α and grown in Luria Bertani broth (LB) with appropriate antibiotic selection.

**Table 1:** **Bacterial strains used in this study**.

**Strain**	**Parent strain**	**Serotype**	**Mutation**	**Marker^a^**	**References**
D39		2			Avery et al., 1944
ΔPAC	D39	2	Ply/PspA/PspC	Tmp-Tet-Erm	Quin et al., 2007
			Deletion	(10 μg/ml-5 μg/ml-0.3 μg/ml)	
R36A	D39	2	Capsule Mutation		Taylor, 1949
LEK20	D39	2	PspK Addition	Kan (500 μg/ml)	This study
AM1000	D39	2	Capsule Mutation		Magee and Yother, 2001
LEK16	D39	2	Capsule Deletion/PspK Addition	Kan (500 μg/ml)	This study
MNZ67		NESp			Park et al., 2012
LEK05	MNZ67	NESp	PspK Deletion	Spec (300 μg/ml)	This study
LEK07	MNZ67	NESp	Ply Deletion	Tmp (10 μg/ml)	This study
LEK11	MNZ67	NESp	PspK/Ply Deletion	Spec-Tmp (300 μg/ml-10 μg/ml)	This study
EF3030		19F			Andersson et al., 1983
LEK10	EF3030	19F	PspC Deletion	Erm (0.3 μg/ml)	This study
LEK12	EF3030	19F	Capsule Deletion	Spec (300 μg/ml)	This study
LEK14	EF3030	19F	PspK Addition	Kan (500 μg/ml)	This study
LEK15	EF3030	19F	Capsule Deletion/PspK Addition	Spec-Kan (300 μg/ml-500 μg/ml)	This study

a *Tmp-trimethoprim; Tet, Tetracycline; Erm, Erythromycin; Kan, Kanamycin; Spec, Spectinomycin*.

### Genetic manipulations

Gene deletions were made through allelic replacement. Pneumococcal transformations were performed in competence media (THY, 0.2% fresh bovine serum albumin, 0.01% CaCl_2_, and 0.1% glucose). Bacteria were grown in competence media to approximately OD_600_ 0.2, diluted 1:20 in competence media, and stimulated with 2 μg/ml of competence-stimulating peptide 1 (CSP-1) for 12 min before the addition of ~1 μg of DNA (Yother et al., 1986). Pneumococci were incubated for 4 h at 37°C before plating on BA containing appropriate antibiotic selection.

DNA for *pspK* deletion in MNZ67 and *cps* deletion in EF3030 was obtained through polymerase chain reaction (PCR) of genomic DNA isolated from MNZ1131 (Park et al., 2012) with primers DexBF (5′-GACTATCTAGCCAAGCTAGG-3′) and AliAR (5′- CCCTGTACGAGATGTAGTTG -3′). DNA for *ply* deletion was isolated from ΔPly2 (Thornton and McDaniel, 2005) using primers UpstreamPlyF (5′-CTAGCCTTGACA ACTAGCCAATC-3′) and DownstreamPlyR (5′-TGCAAA TAGAAAGTTTCAGCC-3′). Expression of PspK was achieved through ectopic expression using plasmid pABG5. The *pspK* gene was isolated from genomic MNZ67 DNA using primers pABG5 PspKF (5′-GCGGAATTCAT GAATAATAAGAATATCATCCCGATG AG-3′) and pABG5 PspKR (5′-GCGCTGCAGCTAATTTTT ATGTTTAACAAATGGAAGA-3′). Primers contain restriction sites EcoRI and PstI, respectively, indicated by bolded and underlined section of primer. Plasmid pABG5 and *pspK* amplicon was digested with EcoRI (NEB) and PstI (NEB), ligated with T4 DNA ligase (Thermo Scientific) and transformed into DH5α making pABG5::*pspK*. Plasmid verified by sequencing and PspK levels in wildtype MNZ67 and PspK expression mutants were equivalent as determined by flow cytometry and Western blot analysis.

### Adhesion and invasion assays

Adhesion and invasion assays were performed as previously described (Keller et al., 2013). In brief, 24 well-plates were seeded to ~90% confluency with human pharyngeal cell line Detroit 562 or lung cell line A549. Epithelial cells were incubated with 1 × 10^7^ CFU/ml of bacteria suspended in EMEM for 30 min then washed two times with 1X PBS to remove unbound pneumococci. Epithelial cells were trypsinized (100 μl 0.25% Trypsin-EDTA) and plated on BA for enumeration. Invasion assays were performed as above, but epithelial cells were incubated with bacteria for 2 h before being washed two times with 1X PBS. Washed cells were further incubated for 1 hr with EMEM containing 10 μg/ml penicillin and 200 μg/ml streptomycin to kill extracellular bacteria. Epithelial cells were washed and trypsinized as above before enumeration on BA.

### Experimental OM

Experimental OM was performed as previously described (Keller et al., 2014). In brief, young adult chinchillas (*Chinchilla lanigera*, body weight 400–500 g) from Ryerson Chinchilla Ranch were allowed to acclimate for at least 7 days. Otoscopic examination was used to examine the tympanic membrane of all animals before infection. Chinchillas with no visible pathology received 100 μl transbullar injections containing 1 × 10^7^ NESp or 1 × 10^2^ encapsulated pneumococci in 1X PBS containing 0.04% gelatin. Differences in inoculum amounts between NESp and encapsulated pneumococci were calculated in accordance to previous studies that displayed encapsulated pneumococci to be highly invasive causing sepsis and death very rapidly at high inoculums in this model (Forbes et al., 2008). Chinchillas were injected with 1 × 10^4^ of the D39 mutant ΔPAC due to a known reduction of virulence in other animal models (Ogunniyi et al., 2007b). Infections with encapsulated pneumococci were only performed out to 3 days due to significant pathology of these strains. Animals were monitored daily for clinical symptoms (listing, loss of appetite, and response to stimulus or noise) and tympanic membrane was visualized for inflammation immediately following euthanasia with a Karl Storz Vetcam XL 692800 20 with an otoscopic attachment. Samples were collected and visible tympanic inflammation and biofilm formation were scored. Tympanic inflammation was scored through otoscopic examination as follows: 0 = none, 1 = inflammation, 2 = effusion, and 3 = tympanic rupture. The tympanic membrane of an uninfected chinchilla is an opalescence off-white color with inflammation defined as visible rubor or yellowing of the membrane. Effusion of the middle ear is indicated by physical presence of fluid upon sample processing or yellowing of the tympanic membrane with visible pockets of air behind the membrane. Tympanic rupture is indicated by a breach of the tympanic membrane with possible drainage. Biofilm formation was visibly scored as follows: 0 = none, 1 = surface formation, 2 = traverses bulla, and 3 = traverses bulla with thickening. For treatment experiments, ampicillin (100 mg/kg) was administered intramuscularly every 12 h starting at the indicated time points. Individual data points in OM studies represent each bulla per animal (2 total), with at least two biological replicates examining 1–2 chinchillas per replicate. All animal studies were performed in accordance with protocols approved by the University of Mississippi Medical Center Institutional Animal Care and Use Committee.

### Murine colonization

Mouse studies were performed as previously described (Keller et al., 2013). In brief, 6–8 week old C57/BL6 mice were lightly anesthetized with isoflurance and intranasally (IN) challenged with 10 μl 1X PBS containing ~1 × 10^7^ bacteria. Five days post-challenge, mice were euthanized, nasopharyngeal lavage, and tissue samples as well as bullae were collected, and bacteria were enumerated on BA containing 5 μg/ml gentamicin. Samples were collected through retrograde lavage of 200 μl sterile saline solution starting from exposed trachea and samples collected from the nares. Mice were decapitated and physically denuded of fur followed by transverse sectioning of skull posterior of orbital sockets. Anterior section of the skull was bisected down sagittal plane and nasopharyngeal tissue collected with forceps and homogenized in 200 μl 1X PBS before enumeration. Colonizing bacteria are a combination of bacterial counts from lavage and nasopharyngeal tissue from the same mouse. Pneumococcal ascension to the middle ear was assessed by bacterial enumeration of bullae collected from the IN challenged mice. The posterior section of the skull was bisected down the sagittal plane and brain removed from both sections. Bullae were located slightly posterior and caudally of external ear canal, removed with forceps, and homogenized in 200 μl 1X PBS before enumeration on BA containing 5 μg/ml gentamicin.

### Statistical analysis

Results from adhesion assays were determined by Student's *t*-test with the InStat program (GraphPad Software). For OM and colonization, the numbers of CFU in the different experimental groups were compared using the Mann-Whitney test with the InStat program. Significant results are indicated by *P* values of <0.05.

## Results

**Figure 1:**
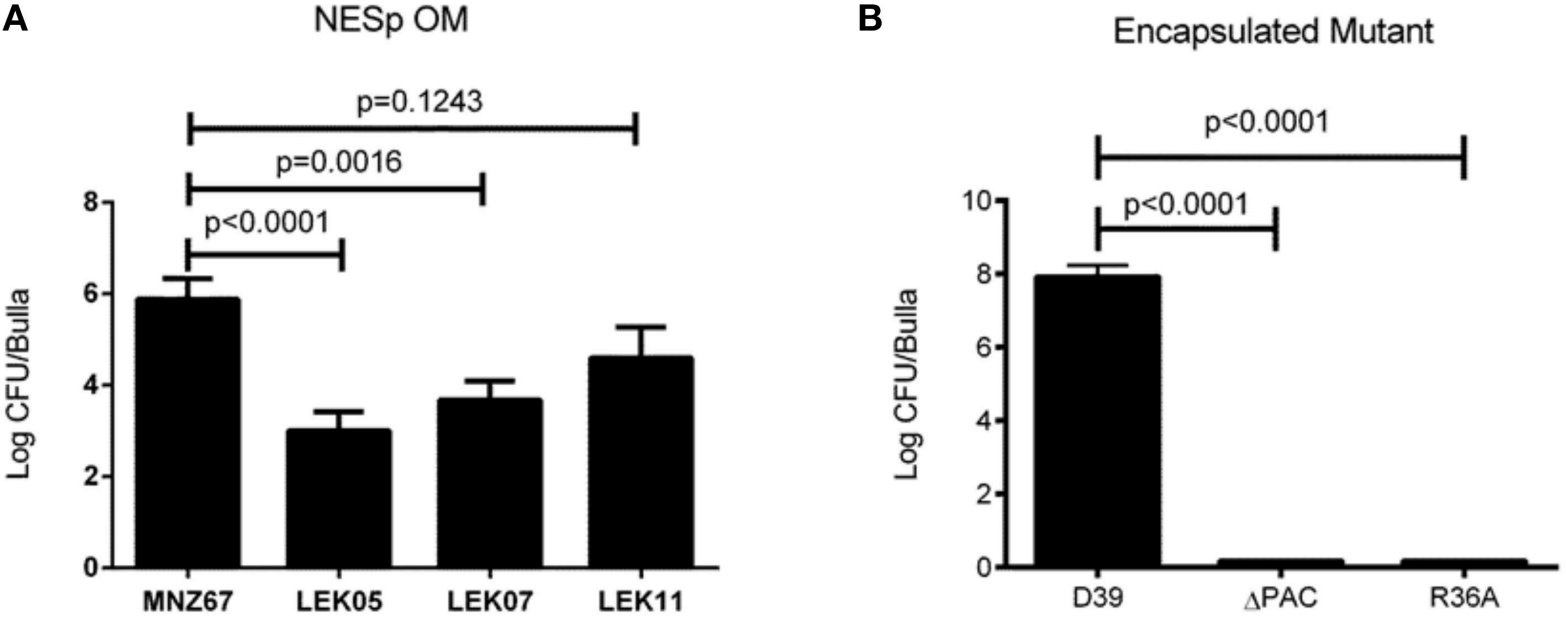
**Deletion of pneumococcal virulence factors significantly reduces bacterial burden during experimental OM. (A)** Single gene deletion of *pspK* (LEK05; *p* < 0.0001) or *ply* (LEK07; *p* = 0.0016) and double gene deletions of *pspK* and *ply* (LEK11; *p* = 0.1243) had significant reductions in recovered bacteria compared to wildtype NESp MNZ67. **(B)** Serotype 2 pneumococcal strain D39 had high levels of bacteria recovered from the middle ear of chinchillas, but no bacteria were recovered when chinchillas were challenged with a D39 triple gene deletion mutant ΔPAC or acapsulalar mutant R36A (*p* < 0.0001). Data represents at least two independent experiments of four bullae per experiment.

We have previously demonstrated that deletion of PspK from NESp MNZ11, a sequence type (ST) 6151, reduced pathogenesis in an OM model and significantly reduced epithelial cell adhesion (Keller et al., 2014). Deletion of PspK from NESp MNZ67 (ST1464) also significantly reduced bacterial burden during OM in a chinchilla model, *p* < 0.0001 (Figure 1A). Inflammation and biofilm formation were significantly reduced when comparing PspK deletion mutant (LEK05) to wildtype (MNZ67), *p* = 0.028 and *p* = 0.0027, respectively (Table 2). Next, the significance of Ply in NESp OM pathogenesis was determined because pneumolysin has been associated with increased inflammation (Hirst et al., 2004). We observed a significant reduction in recovered bacteria from the middle ear (Figure 1A). Also, a significant reduction in biofilm formation was observed when *ply* was deleted (Table 2). Next a double mutant lacking both PspK and Ply was tested for virulence during OM. Surprisingly, there was no significant difference in the number of bacteria recovered from the chinchilla middle ear in comparison to wildtype MNZ67, *p* > 0.05. (Figure 1A).

**Table 2:** **Inflammation and biofilm scores of infected chinchillas**.

**Strain**	**Inflammation score**	**Biofilm formation**	**Inflammation *P*-value**	**Biofilm *P*-value**
MNZ67	1.00 ± 0.41	2.00 ± 0.41		
LEK05	0.00 ± 0.00	0.25 ± 0.25	**0.0286**	**0.0002**
LEK07	0.50 ± 0.29	0.50 ± 0.29	0.33	**0.0015**
LEK11	1.375 ± 0.26	1.00 ± 0.27	0.45	0.061
D39	1.50 ± 0.29	2.00 ± 0.00		
ΔPAC	0.00 ± 0.00	0.00 ± 0.00	**0.0021**	**0.0001**
R36A	0.50 ± 0.25	0.60 ± 0.37	**0.04**	**0.0091**
EF3030	2.00 ± 0.00	1.75 ± 0.25		
LEK10	1.50 ± 0.29	2.00 ± 0.00	0.1354	0.4816

*Scores are average with standard error of the mean with significant p-values indicated in bold. P-value is for mutant vs. parent strain*.

Previous studies have shown the importance of PspA and PspC for encapsulated pneumococci (serotype 2) during OM (Schachern et al., 2014). We have shown the importance of Ply for NESp OM virulence in contrast to past work in encapsulated pneumococci (serotype 3) (Sato et al., 1996). A combination of PspA, PspC, and Ply deletion have not been tested in encapsulated strains. We found that deleting all three proteins from serotype 2 strain D39 (ΔPAC) and deletion of capsule (R36A) completely attenuated virulence in comparison to wildtype, *p* < 0.0001 (Figure 1B). A corresponding reduction in inflammation and biofilm formation was observed when comparing triple mutant ΔPAC and capsule mutant R36A to wildtype D39 *p* < 0.0001 (Table 2).

**Figure 2:**
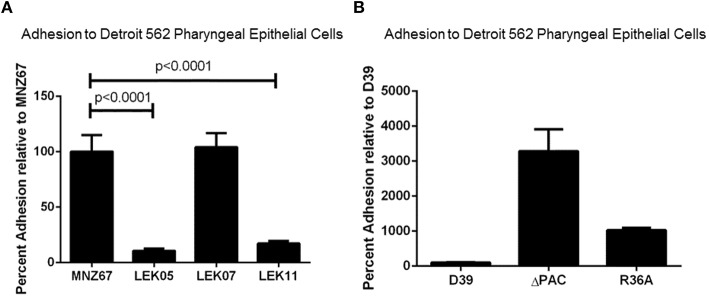
**Adhesion of pneumococci to human pharyngeal epithelial cell line Detroit 562 is affected by specific gene deletions. (A)** Deletion of *pspK* from wildtype NESp MNZ67 significantly reduced bacterial adhesion to epithelial cells, but no effect was seen with a *ply* gene deletion. Deletion of *pspK* and *ply* from MNZ67 (LEK11) significantly reduced epithelial cell adhesion compared to wildtype MNZ67 (*p* < 0.0001). **(B)** Pneumococcal epithelial cell adhesion was significantly increased in both D39 mutants, ΔPAC and R36A (*p* < 0.0001). Data represents three independent experiments in triplicate.

As previously reported, increased adhesion of NESp to epithelial cells correlated with increased bacterial recovery from the middle ear of chinchillas (Keller et al., 2014). Due to reduced bacterial recovery of Ply deletion mutants, we wanted to determine if Ply impacted epithelial adhesion. Although we observed a reduction in pneumococci recovered during OM, Figure 2A shows deletion of *ply* did not reduce epithelial cell adhesion to pharyngeal cell line Detroit 562 (*p* > 0.05). This indicated that another virulence mechanism, such as biofilm formation, may be responsible for the decrease in recovered bacteria. As expected deletion of PspK from wildtype strain MNZ67 did reduce epithelial adhesion (*p* < 0.0001). Deletion of capsule has been shown to increase epithelial cell adhesion of encapsulated strains, which we verified here (Figure 2B). Increased epithelial cell adherence of NESp has led to increased bacterial recovery during experimental OM, but we did not observe that correlation when capsule genes were deleted from encapsulated strains despite increased epithelial cell adherence.

**Figure 3:**
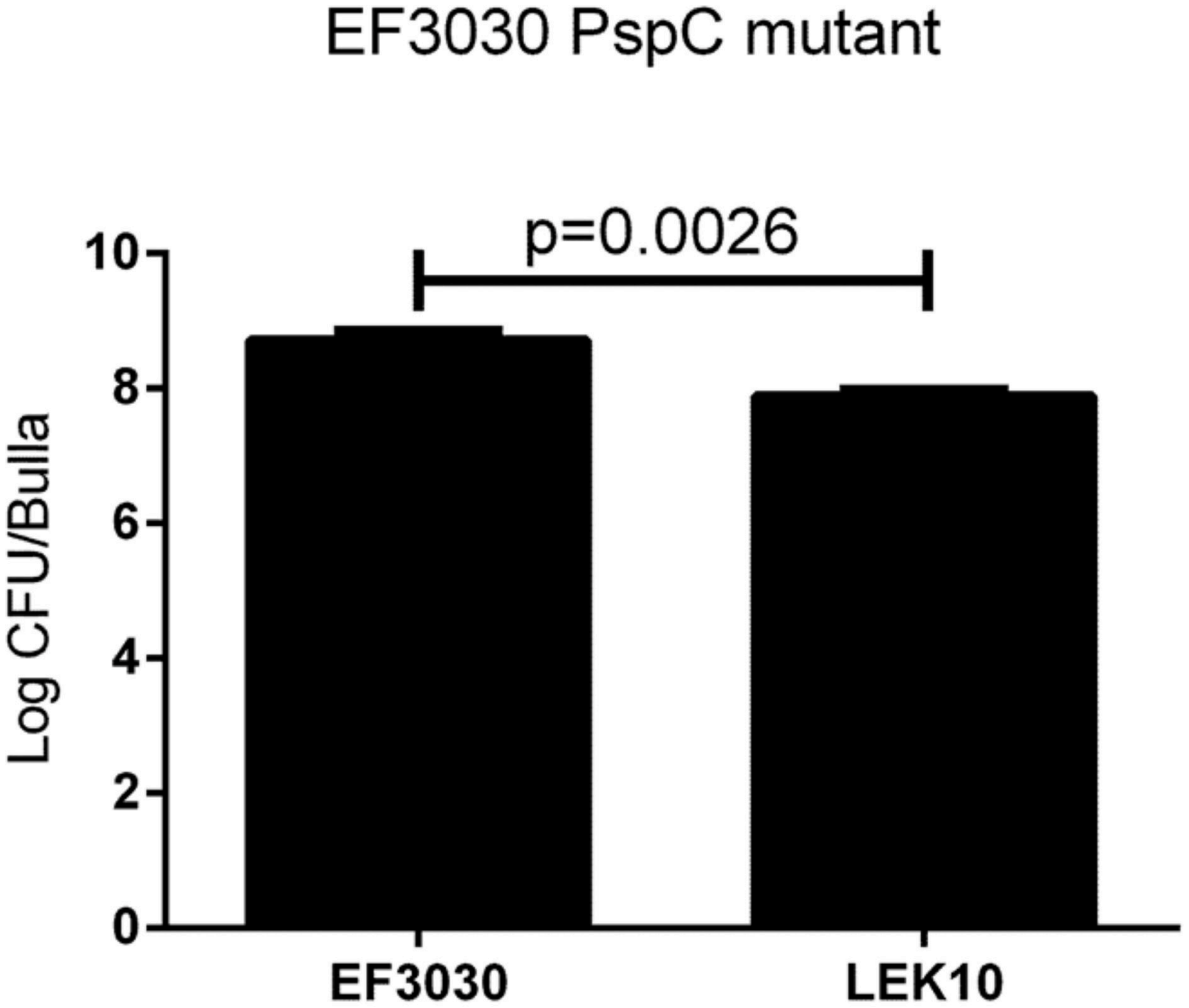
**Encapsulated strain EF3030 (serotype 19F) middle ear pathogenesis in chinchillas are not mediated by PspC**. There is a significant decrease in the amount of bacteria recovered from the middle ear of chinchillas when comparing wildtype EF3030 to *pspC* gene deletion (LEK10). Data represents samples from four bullae.

We have shown that D39 (serotype 2) can infect chinchillas and specific virulence factors play a role in pathogenesis. Unfortunately D39 resulted in high virulence in the chinchilla OM model with as few as 10 CFU causing death in some chinchillas limiting its usefulness due to ethical reasons (data not shown). We wanted to determine if other serotypes were as virulent as D39 during an OM infection, so we investigated encapsulated strain EF3030 (19F, known causative serotype of OM) for our infection model (Joloba et al., 2001). High bacterial loads of EF3030 were recovered from the middle ear of chinchillas during experimental OM (Figure 3) with a portion of infected chinchillas becoming septic. However, chinchillas infected with EF3030 had better clinical outcomes than D39 infected animals (data not shown). No difference between inflammation or biofilm formation was observed between D39 and EF3030, *p* > 0.05 (Table 2). In an effort to reduce invasiveness of encapsulated EF3030, PspC was deleted. Deletion of PspC from EF3030 (LEK10) reduced bacterial burden, *p* = 0.0026, but did not prevent invasive infections in chinchillas (Figure 3).

**Figure 4:**
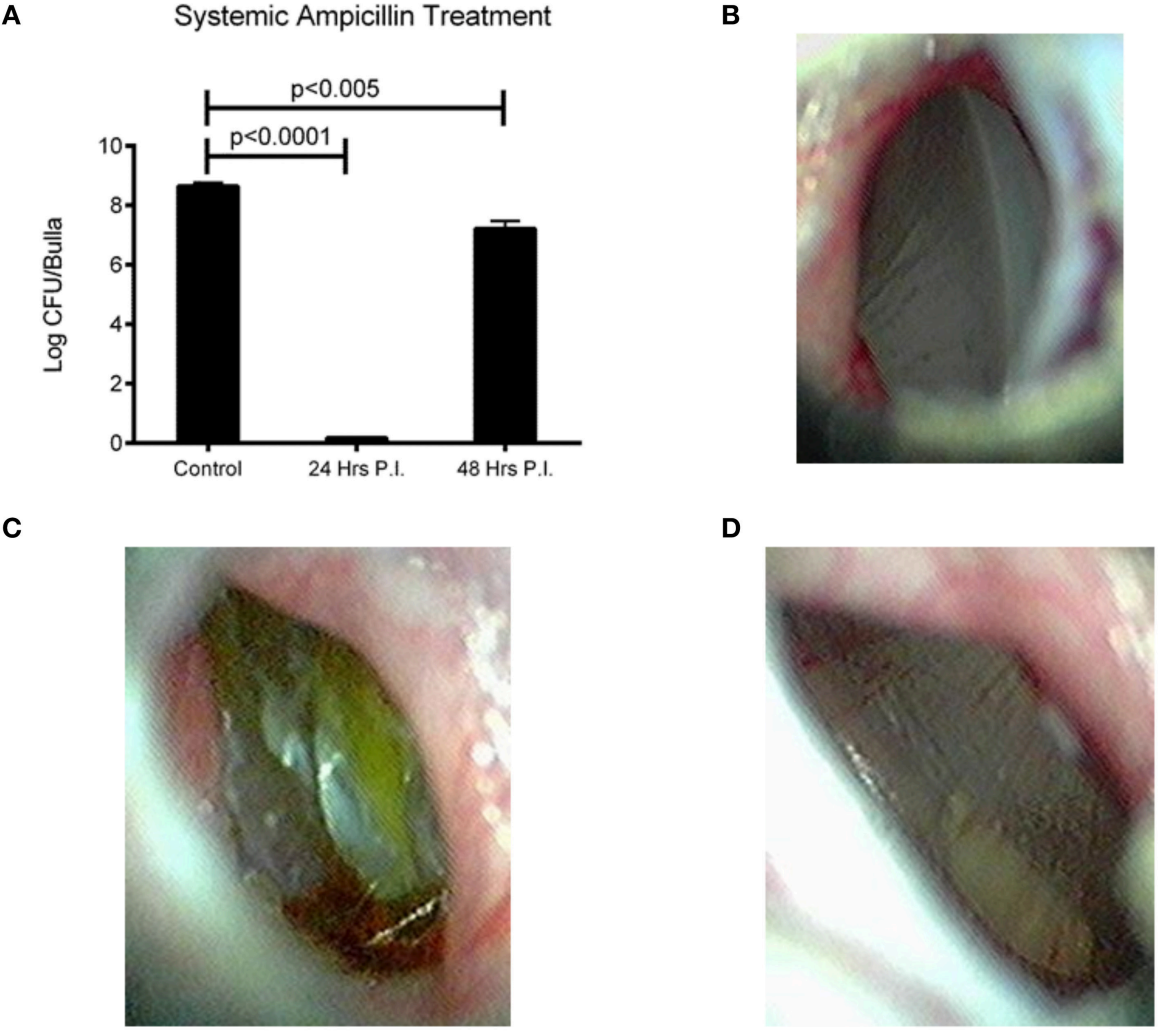
**Effect of bacterial burden and clinical symptoms of systemic ampicillin treatment of EF3030 OM model. (A)** Untreated animals had high levels of recovered bacteria and ampicillin treatment significantly reduced bacterial burden. Treatment starting 24 h post-infection cleared all detectable bacteria from the middle ear and treatment starting 48 h post-infection had a smaller, but significant reduction in recovered bacteria. **(B)** Tympanic membrane of animal 72 h post-infection and 48 h after start of treatment. **(C)** Tympanic membrane of mock treated animal 72 h post-infection. **(D)** Tympanic membrane of animal 72 h post-infection and 24 h after start of treatment. Data represents samples from four bullae and representative pictures.

We next wanted to determine if treatment of an OM infection prone to systemic invasion was treatable with commonly prescribed antibiotics, and if the time of treatment aided in outcome. Amoxicillin is a commonly prescribed antibiotic for the treatment of OM (Lieberthal et al., 2013). The use of ampicillin for our study avoids excess stress upon the animals by reducing the handling necessary to gavage treatment into an infected chinchilla. We found that treatment starting within 24 h post-infection was able to completely clear EF3030 infection, *p* < 0.0001 (Figure 4A). Treatment starting 48 h after infection was also able to reduce bacterial burden, but only by two logs compared to no treatment, *p* < 0.005 (Figure 4A). EF3030 with no treatment had increased inflammation (Figure 4C) compared to treatment starting at 24 h post-infection (Figure 4B) or 48 h post-infection (Figure 4D).

**Figure 5:**
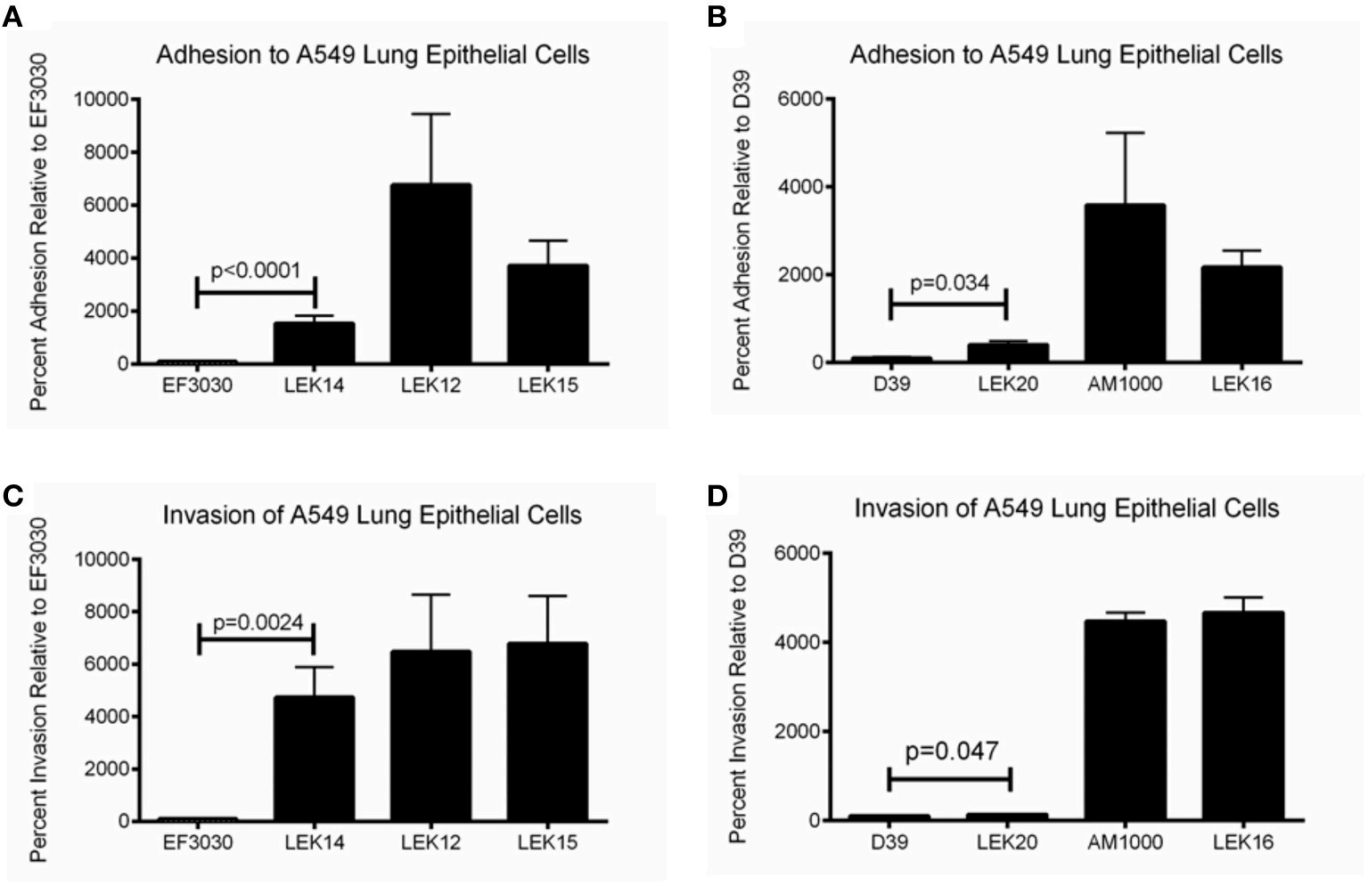
**Encapsulated strain EF3030 (serotype 19F) and mutants adherence and invasion to lung epithelial cell line A549. (A)** Expression of PspK in serotype 19F EF3030 (LEK14) significantly increased epithelial cell adhesion when capsule was present (*p* < 0.0001). Deletion of capsule significantly increased epithelial cell adhesion (LEK12; *p* < 0.0001) compared to wildtype EF3030. Expression of PspK in LEK12 (EF3030Δ*cps*) did not increase adhesion of LEK15 (EF3030Δ*cps*::PspK^+^) compared to acapsular EF3030 (LEK12). **(B)** Expression of PspK in serotype 2 D39 (LEK20) significantly increased epithelial cell adhesion when capsule was present (*p* = 0.034). Deletion of capsule significantly increased epithelial cell adhesion (AM1000; *p* < 0.0001), but expression of PspK in AM1000 (D39Δ*cps*) did not increase adhesion of LEK16 (D39Δ*cps*::PspK^+^). **(C)** Expression of PspK in serotype 19F EF3030 (LEK14) significantly increased epithelial cell invasion when capsule was present (*p* = 0.0024). Deletion of capsule significantly increased epithelial cell invasion (LEK12; *p* = 0.0149) compared to wildtype EF3030. Expression of PspK in LEK12 (EF3030Δ*cps*) did not increase invasion of LEK15 (EF3030Δ*cps*::PspK^+^) compared to acapsular EF3030 (LEK12). **(D)** Expression of PspK in serotype 2 D39 (LEK20) significantly increased epithelial cell invasion when capsule was present (*p* = 0.047). Deletion of capsule significantly increased epithelial cell adhesion (AM1000; *p* < 0.0001), but expression of PspK in AM1000 (D39Δ*cps*) did not increase adhesion of LEK16 (D39Δ*cps*::PspK^+^). Data represents three independent experiments in triplicate.

We have shown that pneumolysin and surface proteins impact pneumococcal virulence and rapid treatment is necessary to efficiently clear infections and limit systemic dissemination. Genetic exchange between pneumococci occurs regardless of capsule status, but the effect of a NESp-specific surface protein expressed in an encapsulated strain has never been determined. Past studies have utilized D39 acapsular derivative R36A for PspK expression in an encapsulated background (Keller et al., 2013, 2014), but R36A has been lab adapted for decades and may not represent a capsule exchange that occurs naturally. Here, we use a PspK expressing plasmid to determine the effects of PspK expression on encapsulated pneumococcal adherence and colonization with and without the presence of capsule. As shown in Figures 5A,B, expression of PspK in encapsulated strains EF3030 and D39 increased their ability to adhere to lung epithelial cell line A549, *p* < 0.0001 and *p* = 0.032 respectively. While this is independent of the two serotypes tested, EF3030 (serotype 19F) had a significantly greater increase in lung epithelial cell adhesion than D39 (serotype 2). The deletion of capsule increased epithelial cell adhesion with or without the presence of PspK. Surprisingly, expression of PspK in acapsular mutants did not significantly increase epithelial cell adhesion when the capsule was absent. This is in contrast to past work using the D39 capsule mutant R36A (Keller et al., 2013). There was a concurrent increase in epithelial cell invasion in all samples in which epithelial cell adhesion was increased (Figures 5C,D).

**Figure 6:**
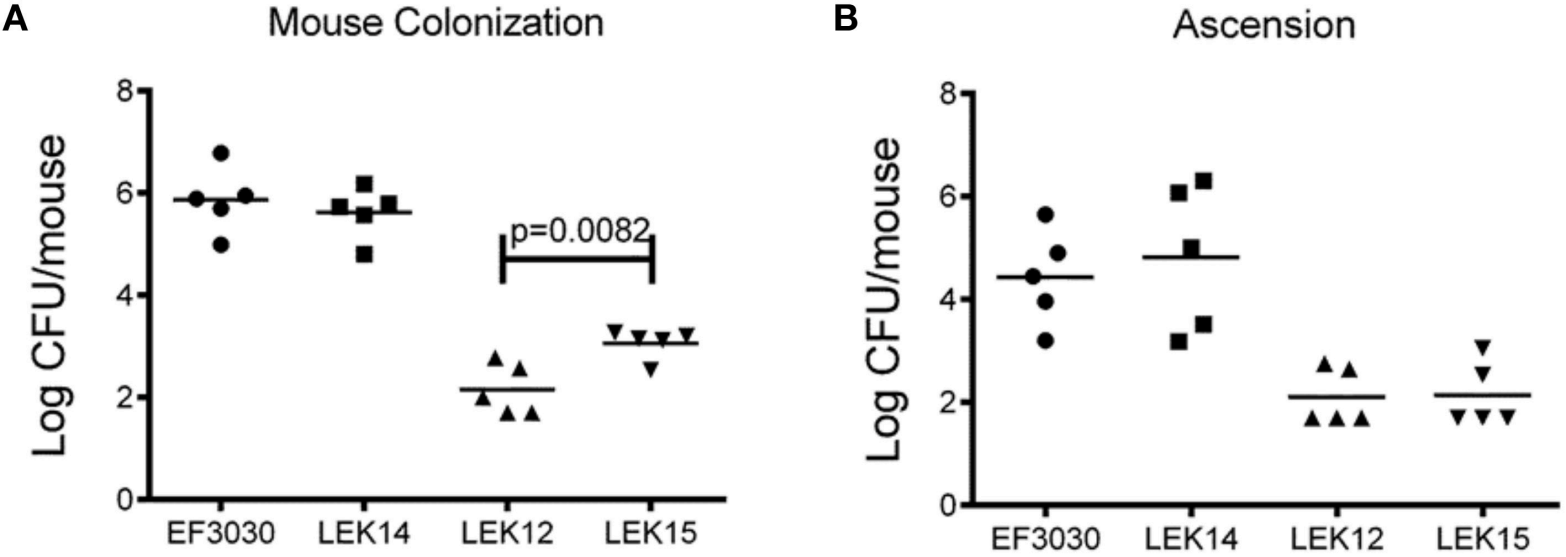
**Encapsulated strain EF3030 (serotype 19F) and mutants in a mouse model of colonization. (A)** At 5 days post-infection, enumerated bacteria recovered from the mouse nasopharynx did not significantly increase when encapsulated EF3030 expressed PspK. EF3030 capsule mutant, LEK12, showed significantly more colonization when PspK was expressed (LEK15; *p* = 0.0082). **(B)** Collected bullae from colonized mice showed no significant difference in middle ear ascension when PspK was expressed independent of capsule status. Mean CFU/mouse indicated by horizontal bar on figure.

We next wanted to determine if these effects *in vitro* were also observed in a mouse model. Since increased adherence may correlate to enhanced virulence of encapsulated strains, the chinchilla OM virulence model was avoided due to ethical consideration of the animals. EF3030 was chosen for this model because it is a known colonizer of the mouse nasopharynx and, unlike D39, does not disseminate into the blood in mice. As shown in Figure 6A, there was no difference in the ability of EF3030 to colonize when PspK was present or absent. In contrast, when the capsule is removed from EF3030 and PspK is expressed, there were significantly more bacteria recovered from the mouse nasopharynx (*p* = 0.0082), but not equivalent to wildtype EF3030 levels. The amount of bacteria that ascended through the mouse Eustachian tube into the middle ear was also quantified (Figure 6B). Expression of PspK in EF3030 or in EF3030 capsule mutant LEK12 did not significantly increase the number of bacteria collected from the middle ear.

## Discussion

PspK plays an important role in NESp pathogenesis along with pneumolysin (Ply) (Figure 1). While PspK mediated virulence is through increased epithelial cell adhesion, Ply virulence is not (Figure 2A). The expression of PspK in encapsulated strains increases adhesion *in vitro* to human epithelial cells (Figures 5A,B), but does not correlate to increased ability to colonize the mouse nasopharynx. Colonization by a capsule mutant of an encapsulated strain is partially rescued by the expression of PspK, but not to the level of a wildtype encapsulated strain (Figure 6A). We were also able to show the importance of surface proteins for OM in encapsulated strains, as well as an increased efficacy of treatment when the treatment is started early during infection.

We first described PspK as a virulence protein in the chinchilla using MNZ11, a ST6151 strain that is genetically related to other NESp based on sequence type (Keller et al., 2014). The current study uses a NESp MNZ67, a ST1464 that has been previously associated with pneumococci of the 19F serotype. We determined that PspK performs a similar function in NESp regardless of the genetic background in which it is being expressed, both in terms of *in vitro* epithelial cell adhesion and *in vivo* pathogenesis. The role of Ply, a known virulence factor found in all encapsulated and nonencapsulated pneumococci, was also examined in NESp virulence. We were able to demonstrate a significant decrease in virulence during OM when Ply was not expressed (Figure 1), and this is independent of epithelial cell adhesion. Decreased virulence was most notable in the amount of biofilm in the middle ear, with the Ply mutant producing significantly less biofilm than wildtype MNZ67 (Table 2). This is unsurprising due to previous reports on the importance of Ply for biofilm formation and helps explain the decrease in recovered bacteria (Shak et al., 2013). Recent reports of NESp have also identified a second Ply-like gene (*plyB*) that is more closely related to cytolysins of other streptococcal species (Morales et al., 2015). The presence of this second cytolysin may compensate for the deletion of the canonical Ply, but this has yet to be tested. This led to the question of the importance of pneumolysin in encapsulated strains during experimental OM.

In contrast to this study with a pneumolysin mutant in NESp, a previous study in encapsulated pneumococci showed no effect on OM virulence (Sato et al., 1996). It has been previously reported that the choline binding proteins PspA and PspC both play a role in pneumococcal pathogenesis, including middle ear infections (Schachern et al., 2014). We examined the encapsulated strain D39 and a triple deletion mutant (ΔPAC) that lacks PspA, PspC, and Ply because previous studies showed variable effects of individual mutants, but none containing all deletions (Schachern et al., 2014). The ΔPAC mutant has been found to be avirulent in other infection models (Ogunniyi et al., 2007b; Quin et al., 2007), but has not been previously tested in chinchillas. NESp lack both PspA and PspC yet are able to cause OM. Additionally, we have shown here that although virulence is reduced, a NESp Ply mutant is still able to cause an infection (Figure 1). The surface of the D39 mutant ΔPAC is similar to NESp by lacking these proteins, but still retains the important capsule virulence factor. Unlike NESp, ΔPAC was unable to cause OM and had no visible signs of pathology. While ΔPAC was inoculated at a lower CFU/ml than NESp, we have been able to previously recover NESp from chinchillas infected with as little as 1 × 10^4^ inoculum but with a reduction in total number of infected animals (unpublished data). While capsule is important for encapsulated pneumococcal pathogenesis, it in itself is not sufficient to cause disease. Surprisingly, ΔPAC had increased adherence in comparison to wildtype D39. While increased adherence of NESp has been associated with higher rates of virulence in an OM model, this does not seem to hold for capsule mutants (Keller et al., 2014). Park et al. (2012) showed significantly less colonization by AM1000 in a mouse model compared to D39 despite higher rates of epithelial cell adhesion. We also examined the encapsulated strain EF3030 (serotype 19F) in a chinchilla model because it is less virulent in a mouse model and the serotype 2 D39 caused fatal pathology in several chinchillas within the time course of the study. We also tested a PspC mutant in the EF3030 background (LEK10) to reduce the invasive nature of EF3030. Both the wildtype and the PspC mutant were able to cause OM, but there was no significant difference between clinical symptoms. However, there was significantly fewer pneumococci recovered from the middle ear of chinchillas infected with the PspC mutant (Figure 3). In both EF3030 and LEK10, we were able to recover bacteria from the blood of infected chinchillas, and this led to the question of treatment.

Amoxicillin is a commonly prescribed antibiotic for middle ear infections, and EF3030 is susceptible to ampicillin and its derivations. Amoxicillin is an oral antibiotic that is absorbed through the stomach and must be ingested for effective treatment. This entails restraining and gavaging chinchillas for treatment, inducing high amounts of stress given that treatment is administered every 12 h. Therefore, an injectable form of ampicillin was used as a substitute. We found that treatment administered 24 h after infection was able to completely clear OM within 48 h of start of treatment. This corresponded to reduced tympanic inflammation and a better clinical outcome. We also show that treatment administered 48 h after infection is still sufficient to reduce the bacterial burden during OM, even though only 24 h had elapsed since start of treatment (Figure 4). It was not surprising that ampicillin treatment cleared the pneumococcal infection, but it was somewhat surprising as how fast this occurred. This information is important to know as studies on treatment of NESp in middle ear infections have not been done, even though these bacteria tend to be highly drug resistant. Additionally, OM co-infections of pneumococcus and nontypeable *Haemophilus influenzae* (NTHi) are common (Casey and Pichichero, 2004; Dagan et al., 2013). It has been shown that secreted beta-lactamases from NTHi significantly improve survival of pneumococci in the middle ear (Weimer et al., 2011). Elucidating treatment and time to resolution of infection is necessary to determine the effectiveness of various antibiotics and to overcome the consequences of co-infections.

An interesting observation during this study was deletion of PspA, PspC, and Ply abolished D39 virulence even when capsule was present. Surface proteins are important for virulence, but NESp that lack common encapsulated surface proteins cause disease. We wanted to determine the effect of PspK expression on encapsulated strains and whether the expression of PspK in a capsule mutant is able to restore virulence. Despite *pspK* being located at the *cps* locus, it may be possible for an encapsulated variant to express PspK due to pneumococcal natural transformation and high levels of genetic variability. It has been observed that *aliD*, a NESp *cps* locus protein, is found in the *cps* locus of some encapsulated strains (Bentley et al., 2006; Keller et al., 2016). Ectopic expression of PspK in an encapsulated background was able to significantly increase the ability of an encapsulated strain to adhere to both lung and pharyngeal epithelial cell lines. This seems independent of capsule as the same effect was observed in a serotype 2 background (D39) and a serotype 19F background (EF3030). While an increase in adherence was observed in both serotypes, there was a significantly greater increase in adhesion when comparing EF3030 to D39 (Figures 5A,B). It is speculated that the capsule structure may affect the ability of PspK to increase adherence. This is because the type 2 capsule is composed of 6 repeating sugar subunits in comparison to the type 19F capsule which contains 3 repeating sugar subunits, which may alter capsule size and accessibility of PspK to epithelial cells (Bentley et al., 2006).

We were able to confirm that the deletion of the *cps* locus from encapsulated strains significantly increased epithelial cell adhesion of these mutants (Figures 5A,B). Surprisingly, the expression of PspK had no significant effect on epithelial cell adhesion when capsule was not present. This could be explained by a saturation of the epithelial cells, reducing the ability of PspK to interact with the epithelial cells. These findings are in contrast to our past work when expression of PspK in R36A did show a significant increase in epithelial cell adhesion. The capsule mutants made for this study were minimally passed, while the D39 derivative R36A has been passed multiple times and for several decades. This may have altered the surface of R36A compared to a recently made capsule mutant. When a mouse was colonized with these PspK expression strains with or without capsule, an opposite trend of what would be expected based on the adhesion data was observed (Figure 6A). The highly adherent EF3030 expressing PspK (LEK14) had no significant difference in colonization. Yet when PspK was expressed in a EF3030 capsule mutant (LEK15), there was significantly more bacteria recovered from the mouse nasopharynx. This effect must be independent of epithelial cell adhesion, as a capsule mutant was adherent with or without PspK expression. We have previously shown that PspK does bind sIgA and this may aid in colonization. Also, we have shown that PspK may be responsible for modulating the host immune response (Keller et al., 2015). If PspK down regulated innate immune responses, it would allow the more vulnerable capsule mutant to readily persist, which was our observation. The effects of PspK on cytokine expression were mild and may explain why only a small increase in colonizing bacteria was observed (Keller et al., 2015).

Taken together this work shows surface structures and Ply are important for pneumococcal OM in both encapsulated and nonencapsulated pneumococci. Also, deletion of multiple virulence factors seems to have a greater impact on virulence than single gene deletions. While this is not surprising, it emphasizes the importance for protein-based vaccines to contain multiple proteins for both broader and more complete protection. Targeting single proteins may not be sufficient to abolish virulence or clear an infection. NESp lack several of the proteins being developed for a protein vaccine, but PspK has been shown to be a protective immunogen (Keller et al., 2015). As replacement of capsule can be partially compensated for by acquisition of PspK, inclusion of PspK in future vaccine development may be necessary to provide the broadest protection possible.

## Author contributions

LK and LM designed experiments. LK, JB, and HP made strains and performed experiments. LK and LM wrote the paper.

## Funding

Funding was provided in part by institutional funds and a grant from Alcon Research to LSM.

### Conflict of Interest Statement

The authors declare that the research was conducted in the absence of any commercial or financial relationships that could be construed as a potential conflict of interest.
